# A Novel Immune Classification for Predicting Immunotherapy Responsiveness in Patients With Adamantinomatous Craniopharyngioma

**DOI:** 10.3389/fneur.2021.704130

**Published:** 2021-12-13

**Authors:** Feng Yuan, Xiangming Cai, Junhao Zhu, Lei Yuan, Yingshuai Wang, Chao Tang, Zixiang Cong, Chiyuan Ma

**Affiliations:** ^1^Department of Neurosurgery, Affiliated Jinling Hospital, Medical School of Nanjing University, Nanjing, China; ^2^School of Medicine, Southeast University, Nanjing, China; ^3^Department of Anesthesiology, First Affiliated Hospital of Anhui Medical University, Hefei, China; ^4^Department of Internal Medicine III, University Hospital Munich, Ludwig-Maximilians-University Munich, Munich, Germany; ^5^Jinling Hospital of Southern Medical University, Nanjing, China; ^6^School of Medicine, Nanjing Medical University, Nanjing, China

**Keywords:** adamantinomatous craniopharyngioma (ACP), immune microenvironment (IME), classification, immunotherapy, nomogram

## Abstract

Adamantinomatous craniopharyngioma (ACP) is the most common tumor of the sellar region in children. The aggressive behavior of ACP challenges the treatment for it. However, immunotherapy is rarely studied in ACP. In this research, we performed unsupervised cluster analysis on the 725 immune-related genes and arrays of 39 patients with ACP patients in GSE60815 and GSE94349 databases. Two novel immune subtypes were identified, namely immune resistance (IR) subtype and immunogenic (IG) subtype. Interestingly, we found that the ACPs with IG subtype (34.78%, 8/23) were more likely to respond to immunotherapy than the ACPs with IR subtype (6.25%, 1/16) *via* tumor immune dysfunction and exclusion (TIDE) method. Simultaneously, the enrichment analysis indicated that the differentially expressed genes (DEGs) (*p* < 0.01, FDR < 0.01) of the IG subtype were chiefly involved in inflammatory and immune responses. However, the DEGs of the IR subtype were mainly involved in RNA processing. Next, immune infiltration analysis revealed a higher proportion of M2 macrophage in the IG subtype than that in the IR subtype. Compared with the IR subtype, the expression levels of immune checkpoint molecules (PD1, PDL1, PDL2, TIM3, CTLA4, Galectin9, LAG3, and CD86) were significantly upregulated in the IG subtype. The ssGSEA results demonstrated that the biofunction of carcinogenesis in the IG subtype was significantly enriched, such as lymphocyte infiltration, mesenchymal phenotype, stemness maintenance, and tumorigenic cytokines, compared with the IR subtype. Finally, a WDR89 (the DEG between IG and IR subtype)-based nomogram model was constructed to predict the immune classification of ACPs with excellent performance. This predictive model provided a reliable classification assessment tool for clinicians and aids treatment decision-making in the clinic.

## Introduction

Craniopharyngioma (CP) constitutes 1.2–4.6% of all intracranial tumors, accounting for 0.5–2.5 new cases per 1 million population per year globally, of which 30–50% are diagnosed during childhood and adolescence (1–3). The two histological subtypes of CP, adamantinomatous CP (ACP) and papillary CP (PCP) differ in their genesis and age distribution (4). ACP has a bimodal age distribution, with peak incidences in children aged 5–15 years and adults aged 45–60 years. In the childhood and adolescent age group, the APC histological type with cyst formation is the most common. PCPs occurs almost exclusively in adults, at a mean patient age of 40–55 years, and no sex differences have been observed (1, 2, 5, 6).

The current standard treatment for CP is surgery with or without radiotherapy. Although CP is considered histologically benign (WHO grade I), the prognosis and outcomes of CPs are frequently impaired due to the hypothalamus–pituitary location of the CP and tumor-related and/or treatment-related injury to these important structures (7, 8). There is an urgent need for safe and effective alternative therapies to reduce side effects and improve quality of life.

In recent years, cancer immunotherapy has experienced remarkable advances and shifted the paradigm for the treatment of malignancies. Impressive clinical responses have been achieved for several types of solid cancers (such as melanoma, non-small cell lung cancer, and bladder cancer) after treatment with immune checkpoint blockade (ICB) therapy (9). However, cancer immunotherapy is rarely studied in patients with CP.

Through in-depth analysis of the genomic, transcriptomic, and proteomics of patients with ACP, researchers found that the immune response process plays an important role in the pathogenesis of ACP (10). The tumor immune process (or the tumor-immunity cycle) is the basis of immunotherapy and the key to treatment strategies and drug development (11). Therefore, patients with CP have the potential to benefit from cancer immunotherapy.

In this research, we collected a total of 401 samples, including 210 RNA-sequencing data from the GSE68015 database and 110 RNA-sequencing data from the GSE494349 database to investigate the intratumoral immune profile of ACP and explore a novel immune classification for predicting immunotherapy responsiveness. Subsequently, we constructed a gene-based classification prediction model to guide clinical diagnosis and treatment.

## Patients and Methods

### Databases

We collected a total of 401 samples, including 210 RNA-sequencing data from the GSE68015 database and 110 RNA-sequencing data from the GSE494349 database. GSE68015 and GSE94349 databases were downloaded from Gene Expression Omnibus (GEO) (http://www.ncbi.nlm.gov/geo). GSE68015 database (*n* = 210) contains 15 ACP tumor samples, nine normal pituitary tissue samples (controls), 16 normal brain tissue samples, and 170 other primary pediatric and adult brain tumor samples. GSE94349 database (*n* = 191) includes 24 ACP tumor samples, 23 normal pituitary samples, 27 normal brain tissue samples, and 117 surgical tumor samples of other primary pediatric and adult brain tumor types. Gene expression profiles were performed using Affymetrix HG-U133plus2 chips (Platform GPL570).

### Bioinformatic Analysis

ESTIMATE algorithm was applied to calculate the fraction of stromal and immune cells with the R package “estimate” (12). The proportion of tumor-infiltrating immune cell (TIC) was explored using the CIBERSORT algorithm (13). The differentially expressed genes (DEGs) between cluster 1 and cluster 2 groups were determined using a threshold *p*-value of 0.05 by Morpheus online software (https://software.broadinstitute.org/morpheus/) (14). Pearson correlation analysis was applied to identify genes correlated with WDR89 (Pearson |*R*| > 0.5). Gene ontology (GO) and Kyoto Encyclopedia of Genes and Genomes (KEGG) analysis were applied for DEGs and genes that were most correlated with WDR89 (15). We obtained the metagene signatures for angiogenic activity (16), antiapoptotic and proapoptotic (17), tumorigenic cytokines (18), mesenchymal phenotype (19), lymphocyte infiltration (20), proliferation (20), and stemness maintain (21). Single-sample gene set enrichment analysis (ssGSVA) was performed to acquire the enrichment score of each biofunction signature using the “GSVA” R package (22).

### Construction of Immune Classification Predicted Model

The least absolute shrinkage and selection operator (LASSO) method and logistic regression analysis were used to identify the best predictive genes (23). A gene-based nomogram model was constructed to predict the classification of ACPs using the “rms” R package (24).

### Prediction of the Immunotherapy Response

Tumor immune dysfunction and exclusion (TIDE) is a computational method developed in 2018 to predict the ICB response (25). A Bonferroni-corrected *p*-value < 0.05 was considered statistically significant.

### Statistical Analysis

R language (version 3.6.1, http://www.r-project.org) was used as the principal tool for statistical analysis and graphic work.

## Results

### ACPs Classification Based on Immune Infiltration

**Figure 1:**
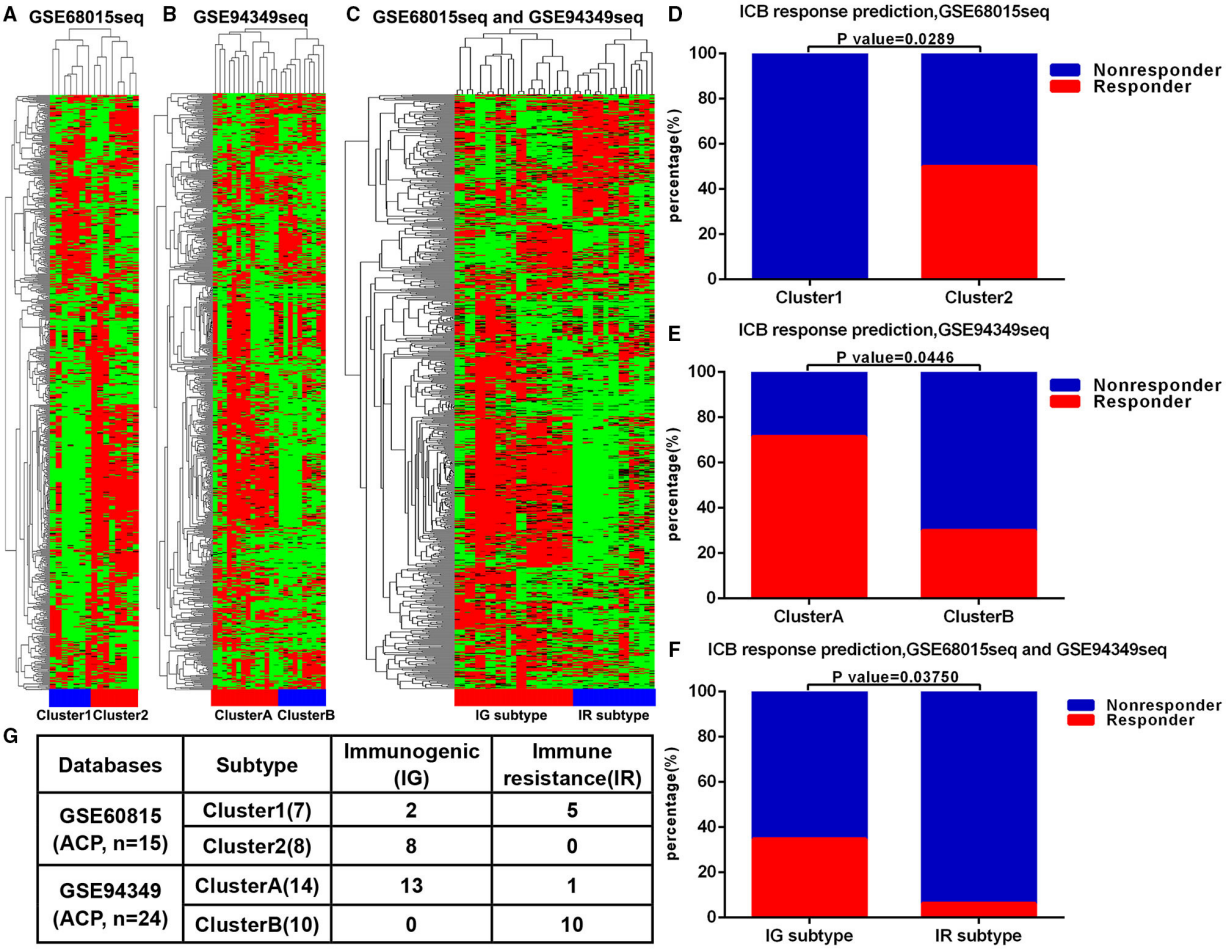
ACPs classification based on immune infiltration. **(A)** The immune microenvironment (IME) of ACPs in the GSE68015 database could be divided into two different clusters, namely cluster 1 and cluster 2. **(B)** The IME of ACPs in the GSE94349 database could also be divided into two different clusters (cluster A and cluster B). **(C,G)** After merging these two databases, ACPs in the cluster 1 group could be matched with ACPs in the cluster B group well, and ACPs in the cluster 2 group could be matched with ACPs in the cluster A group well. **(D–F)** The accumulative bar diagram showed that the ACPs with cluster 2 and cluster A (50%, 4/8; 71.43%, 10/14) were more likely to respond to ICB immunotherapy than the ACPs with cluster 1 and cluster B (0%, 0/7; 30%, 3/10). **(G)** Immune classification and naming of ACPs. ICB, immune checkpoint blockade; IG, immunogenic; IR, immune resistance.

First, we performed unsupervised cluster analysis on the 725 immune related genes and arrays of 15 patients with ACP in the GSE60815 database. The immune microenvironment (IME) of ACPs was divided into two different clusters, namely cluster 1 and cluster 2 (Figure 1A). Similarly, unsupervised cluster analysis was performed on the 725 immune related genes and arrays of 24 patients with ACP in the GSE94349 database, ACPs were also divided into two different clusters (cluster A and cluster B) (Figure 1B). After merging these two databases, we performed usnsupervised cluster analysis on the 725 immune related genes and arrays of 39 patients with ACP again. The results showed that cluster 1 matched well with cluster B, and cluster 2 matched with cluster A (Figures 1C,G). The TIDE results showed that the ACPs with cluster 2 and cluster A (50%, 4/8; 71.43%, 10/14) were more likely to respond to immunotherapy than the ACPs with cluster 1 and cluster B (0%, 0/7; 30%, 3/10) (Figures 1D–F). Therefore, we defined cluster 2/cluster A as the immunogenic (IG) subtype and cluster 1/cluster B as the immune resistance (IR) subtype (Figure 1G).

### Enrichment Analysis in the IG and IR Subtypes of ACPs

**Figure 2:**
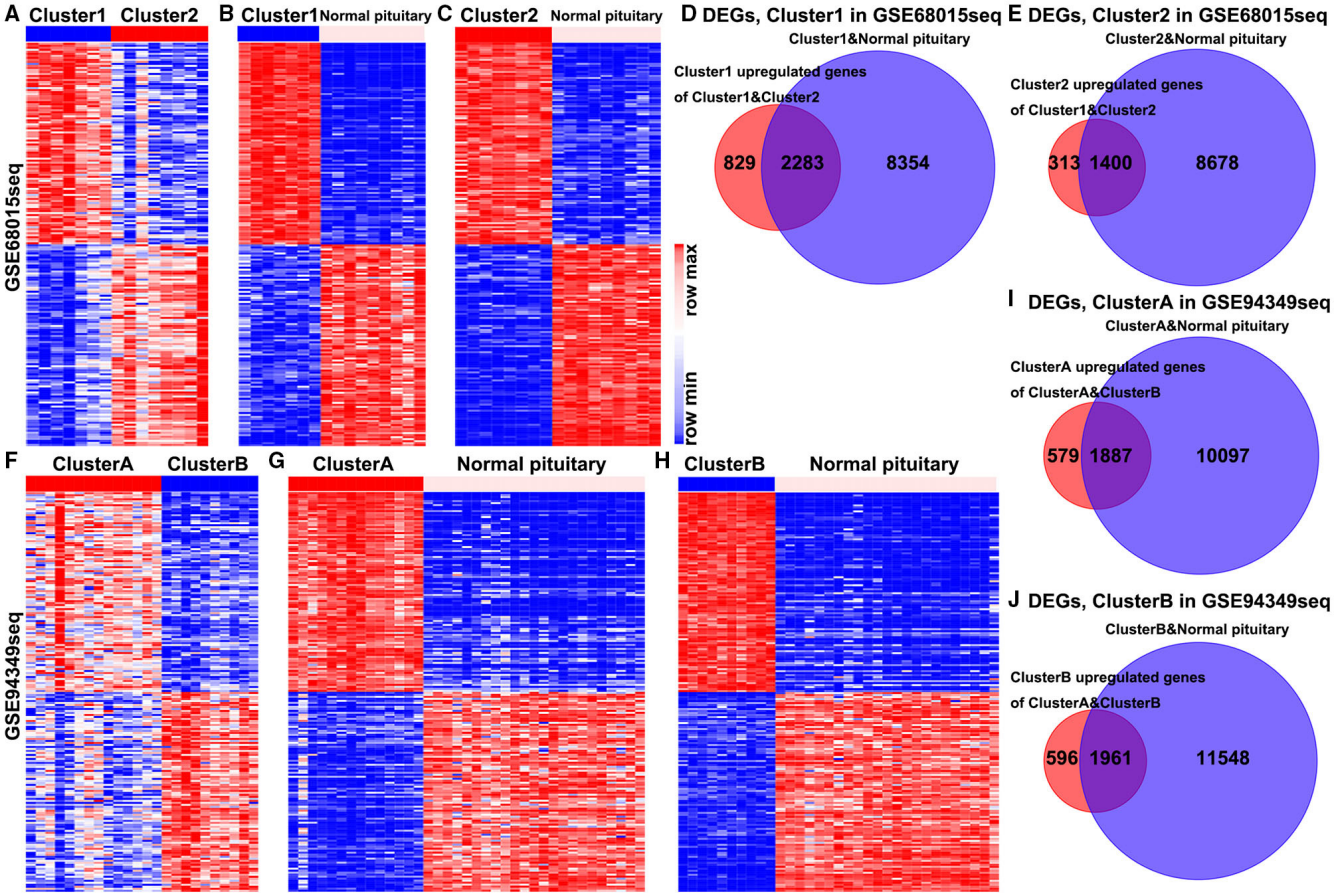
Identification of DEGs in the IG and IR subtypes of ACPs. **(A)** Heat map displaying the DEGs between-group cluster 1 and cluster 2 ACPs in the GSE68015 database. The heat maps display the DEGs between **(B)** cluster 1 and normal pituitary, **(C)** cluster 2 and normal pituitary. **(D)** A total of 3,112 cluster 1 upregulated genes were compared with 10,637 DEGs between the cluster 1 and normal pituitary groups, yielding a set of 2,283 overlapping genes. **(E)** A total of 1,713 cluster 2 upregulated genes were compared with 10,078 DEGs between the cluster 2 and normal pituitary groups, yielding a set of 1,400 overlapping genes. **(F)** Heat map displaying the DEGs between-group cluster A and cluster B ACPs in the GSE94349 database. The heat maps display the DEGs between **(G)** cluster A and normal pituitary, **(H)** cluster B and normal pituitary. **(I)** A total of 2,466 cluster A upregulated genes were compared with 11,984 DEGs between cluster A and normal pituitary groups, yielding a set of 1,887 overlapping genes. **(J)** A total of 2,557 cluster B upregulated genes were compared with 13,509 DEGs between the cluster B and normal pituitary groups, yielding a set of 1,961 overlapping genes.

In the GSE68015 database, we found 4,825 DEGs (3,112 upregulated genes of cluster 1 and 1,713 upregulated genes of cluster 2) between the cluster 1 and the cluster 2 groups (Figure 2A), 10,637 DEGs that were identified between cluster 1 and the normal pituitary groups (Figure 2B) and 10,078 DEGs between the cluster 2 and the normal pituitary groups (Figure 2C). Then, we compared the above-mentioned genes. A total of 2,283 and 1,400 overlapped DEGs specific to the cluster 1 and cluster 2 groups were yielded, respectively (Figures 2D,E). Simultaneously, in the GSE94349 database, we found 5,023 DEGs (2,466 upregulated genes of cluster A and 2,557 upregulated genes of cluster B) between the cluster A and the cluster B groups (Figure 2F), 11,984 DEGs between cluster A and the normal pituitary groups (Figure 2G), and 13,509 DEGs between the cluster B and the normal pituitary groups (Figure 2H). Then, we compared the above-mentioned genes, and a total of 1,887 and 1,961 overlapped DEGs specific to the cluster A and cluster B groups were detected, respectively (Figures 2I,J).

**Figure 3:**
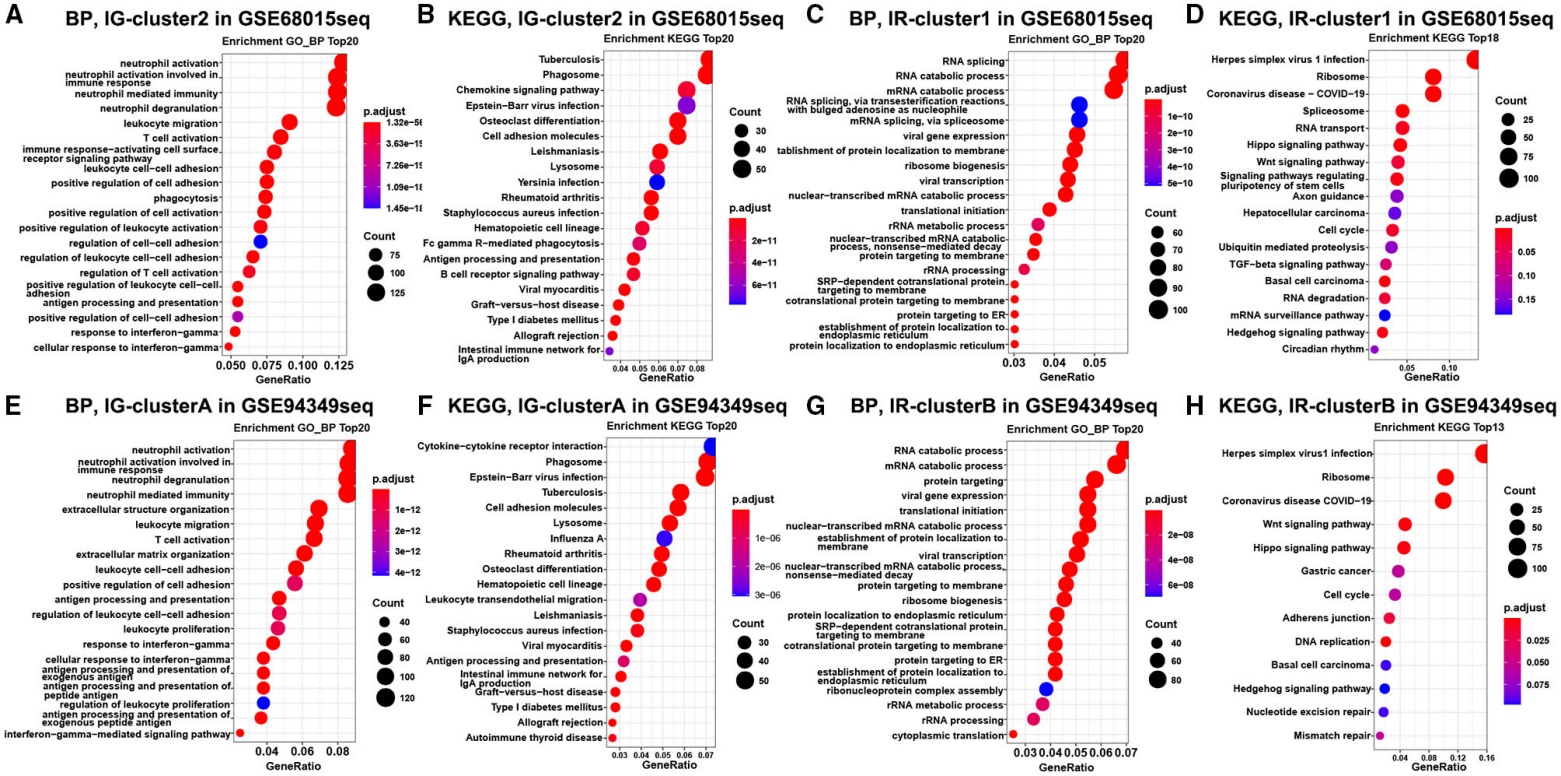
Enrichment analysis in the IG and IR subtypes of ACPs. GO and KEGG enrichment analysis of DEGs in the cluster 1 **(A,B)** and cluster 2 **(C,D)** groups. Enrichment analysis of DEGs in the cluster A **(E,F)** and cluster B **(G,H)** groups. The top 20 items were displayed in the bubble chart.

Finally, the enrichment analysis results indicated that the DEGs of the IG subtype were chiefly involved in various inflammatory and immune responses, as well as a chemokine signaling pathway, antigen processing, and presentation (Figures 3A,B,E,F). However, the DEGs of the IR subtype were mainly involved in RNA splicing, RNA catabolic process, cell cycle, Wnt, and Hippo signaling pathway (Figures 3C,D,G,H).

### Scores in the IG and IR Subtypes of ACPs

**Figure 4:**
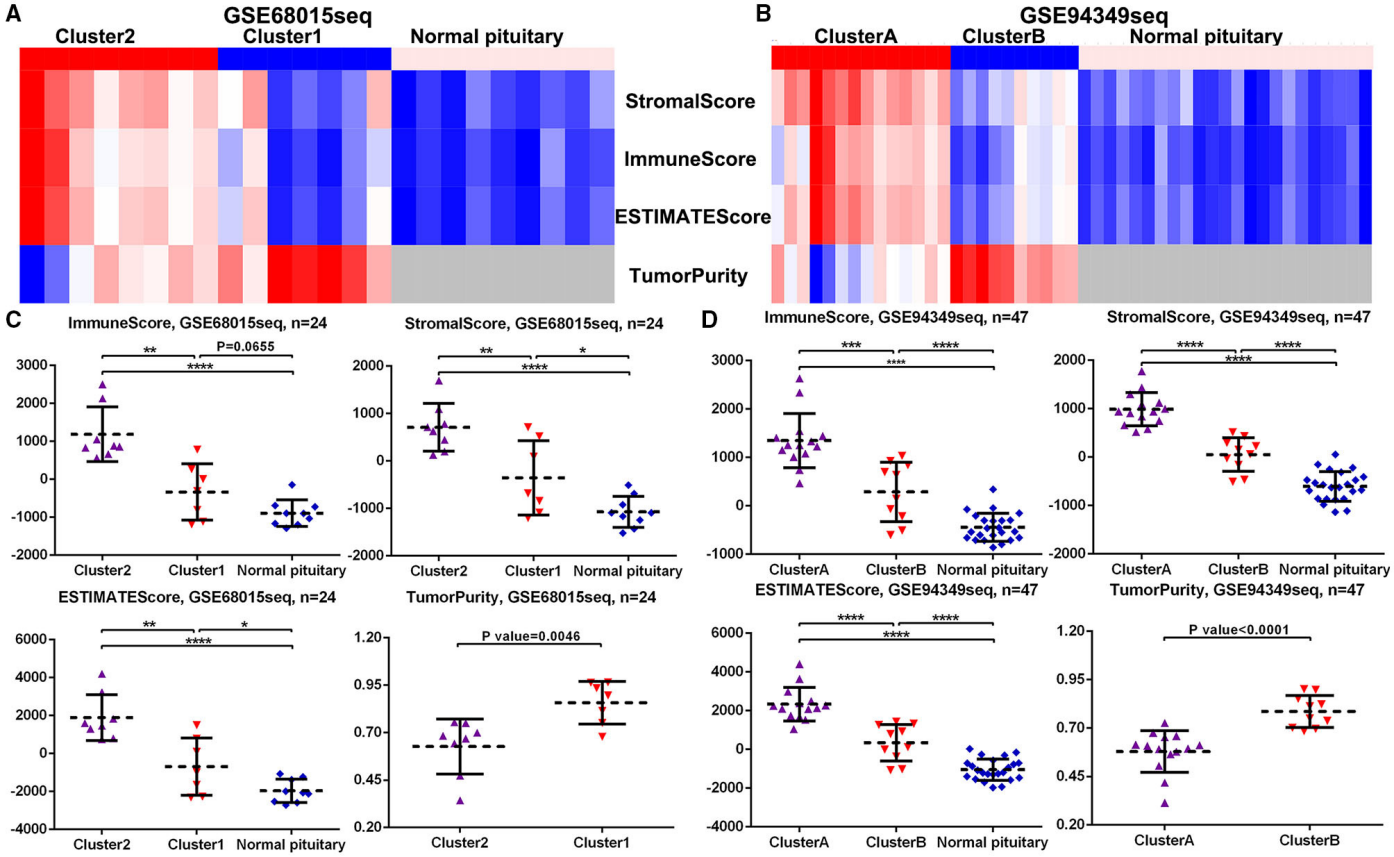
Scores in the IG and IR subtypes of ACPs. **(A,B)** Heat map displaying the distribution of scores among cluster 1/2, cluster A/B and normal pituitary groups in the GSE68015 and GSE94349 databases. **(C,D)** The Scatter plot demonstrated the ratio differentiation of scores and TumorPurity among cluster 1/2, cluster A/B and normal pituitary groups in the GSE68015 and GSE94349 databases. *p < 0.05, **p < 0.01, ***p < 0.001, ****p < 0.0001.

In both GSE60815 and GSE94349 databases, the ESTIMATE results suggested that compared with the normal pituitary group, ACPs had higher immune and stromal scores. In ACPs, compared with IR subtype (cluster 1 and cluster B groups), ACPs in IG subtype (cluster 2 and cluster A groups) had higher immune and stromal scores, while the purity of tumors was lowered (Figures 4A–D).

### The Proportion of TICs in the IG and IR Subtypes of ACPs

**Figure 5:**
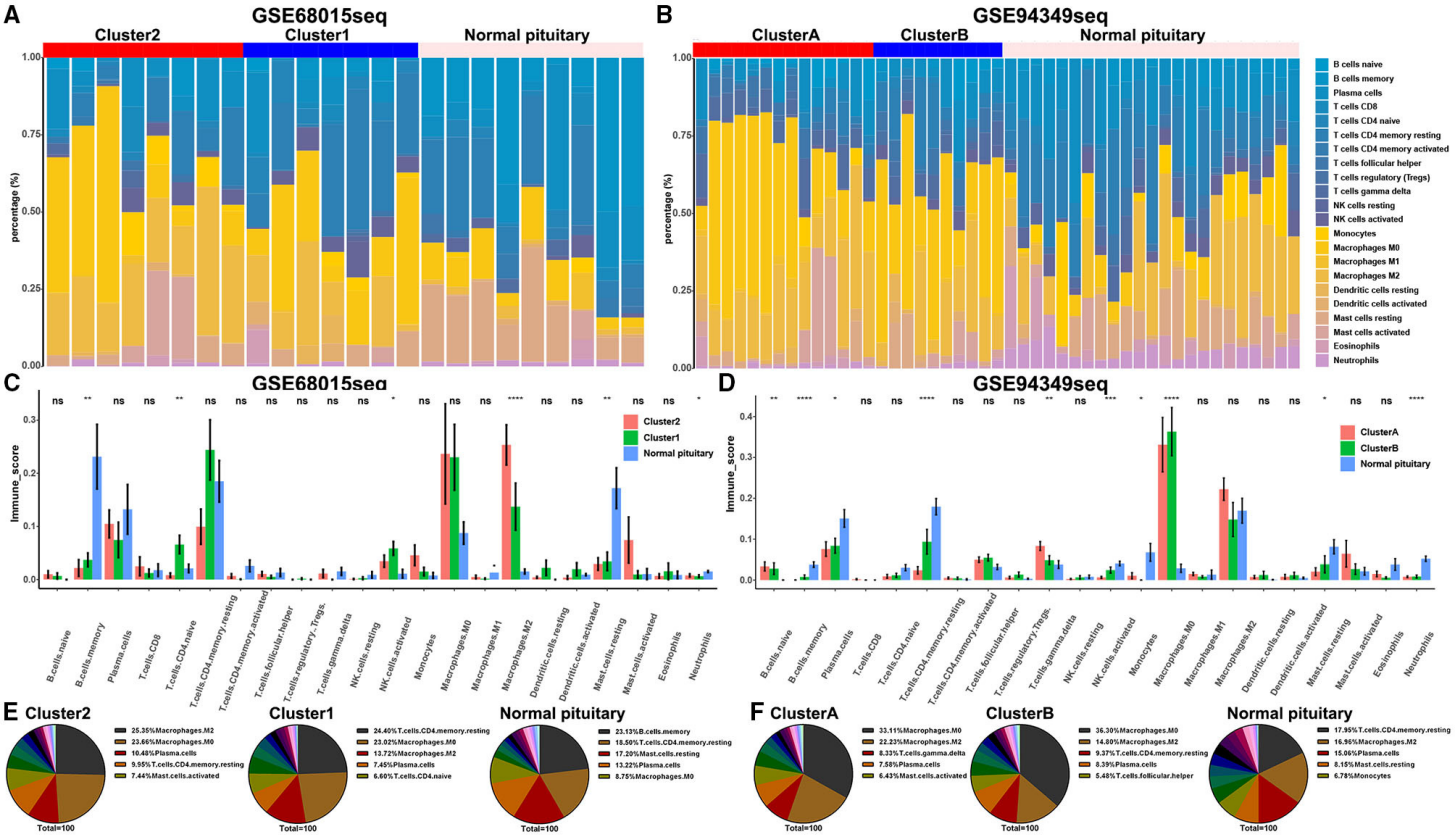
Proportion of TICs in the IG and IR subtypes of ACPs. **(A,B)** Barplot showing the proportion of 21 kinds of TICs in ACPs and normal pituitary in the GSE68015 and GSE94349 databases. Column names of the plot were sample ID. **(C,D)** The bar chart showed the ratio differentiation of 21 kinds of immune cells among cluster 1/2, cluster A/B and normal pituitary groups. **(E,F)** Pie charts showed the distribution of the immune cells among cluster 1/2, cluster A/B and normal pituitary groups. *p < 0.05, **p < 0.01, ***p < 0.001, ****p < 0.0001.

The CITICSORT results found that compared with the normal pituitary group, the proportion of M0 and M2 macrophages was significantly higher in ACPs. The proportion of M2 macrophage in the IG subtype (cluster 2 and cluster A groups) was higher than that in the IR subtype (cluster 1 and cluster B groups). However, the proportion of T cell CD4 memory resting and mast cell resting in the normal pituitary group was distinctly higher than that in the ACPs (Figures 5A–F).

### Expression of Immune Checkpoint Molecules in the IG and IR Subtypes of ACPs

**Figure 6:**
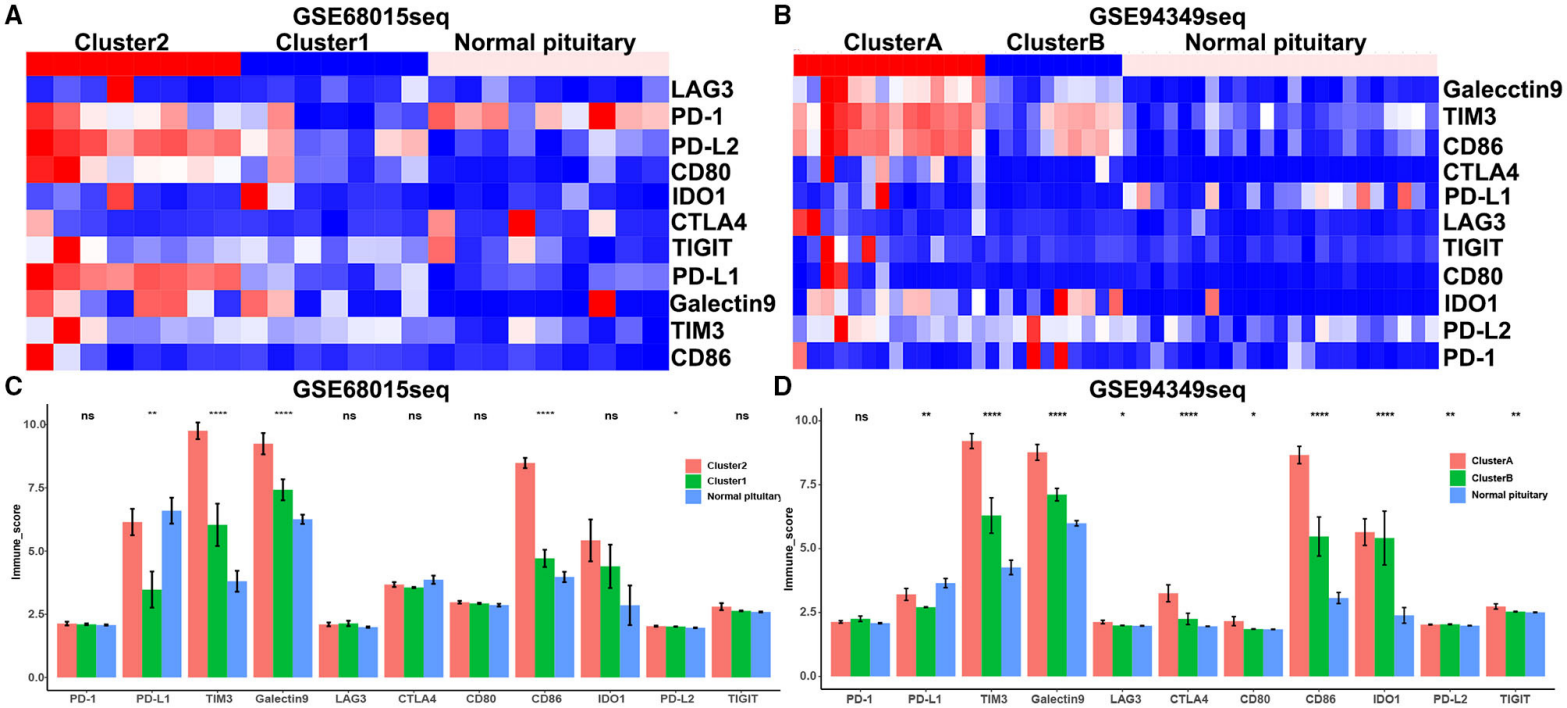
Expression of immune checkpoint molecules in the IG and IR subtypes of ACPs. **(A,B)** Heat map displaying the distribution of immune checkpoint molecules (PD1, PDL1, PDL2, TIM3, CTLA4, Galectin9, LAG3, and CD86) among cluster 1/2, cluster A/B and normal pituitary groups in the GSE68015 and GSE94349 databases. **(C,D)** The bar chart showed the ratio differentiation of immune checkpoint molecules among cluster 1/2, cluster A/B and normal pituitary groups. *p < 0.05,**p < 0.01, ****p < 0.0001.

We also discovered that the expression levels of immune checkpoint molecules (PD1, PDL1, PDL2, TIM3, CTLA4, Galectin9, LAG3, and CD86) were significantly increased in IG subtype (cluster 2 and cluster A groups) compared with IR subtype (cluster 1 and cluster B groups) (Figures 6A–D).

### ssGSVA Between the IG and IR Subtypes of ACPs

**Figure 7:**
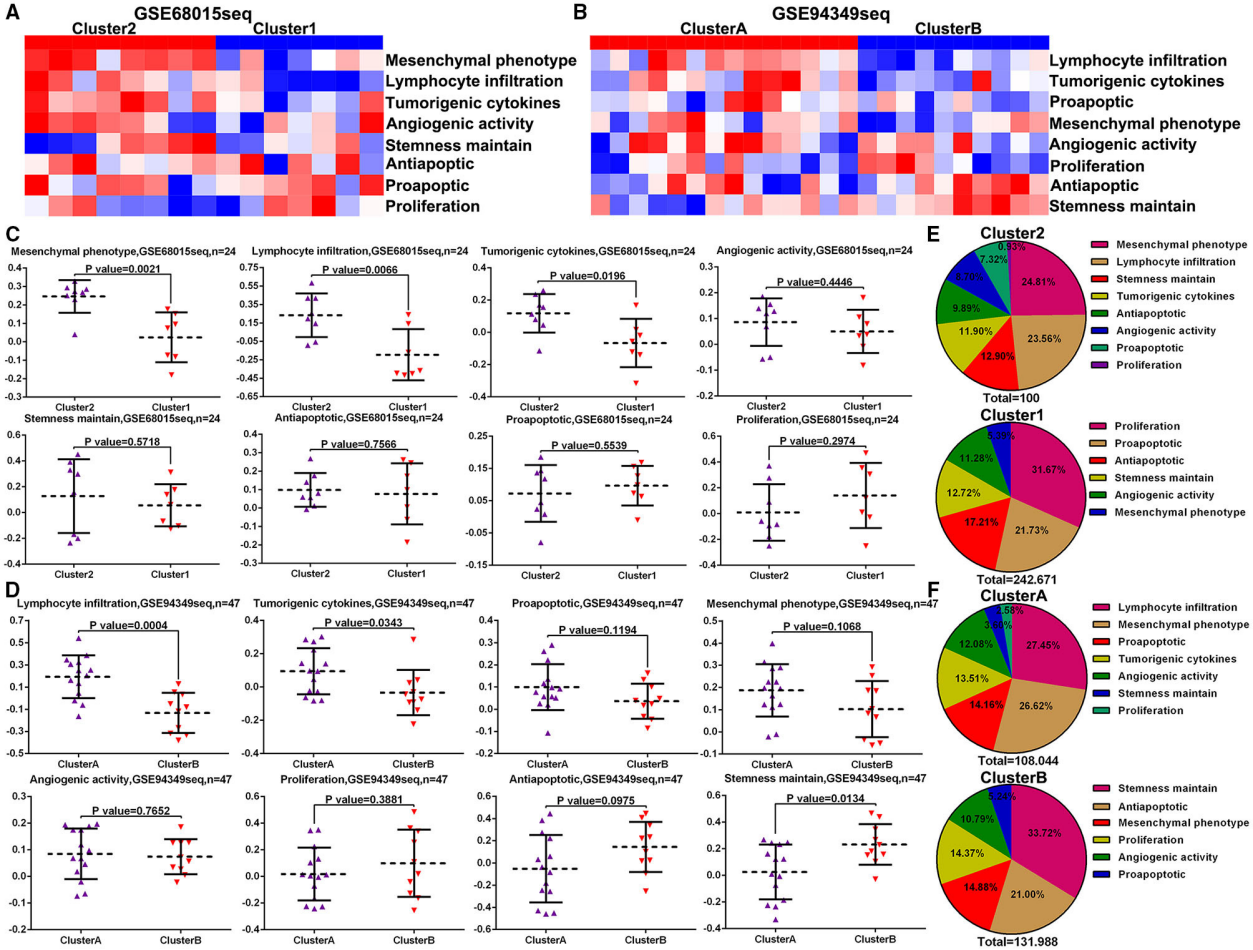
ssGSVA between the IG and IR subtypes of ACPs. **(A,B)** Heat map displaying the distribution of eight biofunctions (proliferation, proapoptic, antiapoptic, stemness maintain, angiogenic activity, tumorigenic cytokines, lymphocyte infiltration, and mesenchymal phenotype) between cluster 1/2 and cluster A/B groups in the GSE68015 and GSE94349 databases. **(C,D)** The Scatter plot indicated the ratio differentiation of eight biofunctions among cluster 1/2, cluster A/B and normal pituitary groups. **(E,F)** Pie charts displayed the distribution of these biofunctions between cluster 1/2 and cluster A/B groups.

To further investigate the different biofunctions between the IG and IR subtypes of ACPs, the ssGSEA analysis demonstrated that the biofunction of carcinogenesis, such as lymphocyte infiltration, mesenchymal phenotype, stemness maintenance, and tumorigenic cytokines, in IG subtype were significantly enriched compared with IR subtype (Figures 7A–D). Moreover, the proportion of lymphocyte infiltration and mesenchymal phenotype in the IG subtype of ACPs was obviously higher than that in the IR subtype of ACPs (Figures 7E,F).

### Construction of the Classification Prediction Model

**Figure 8:**
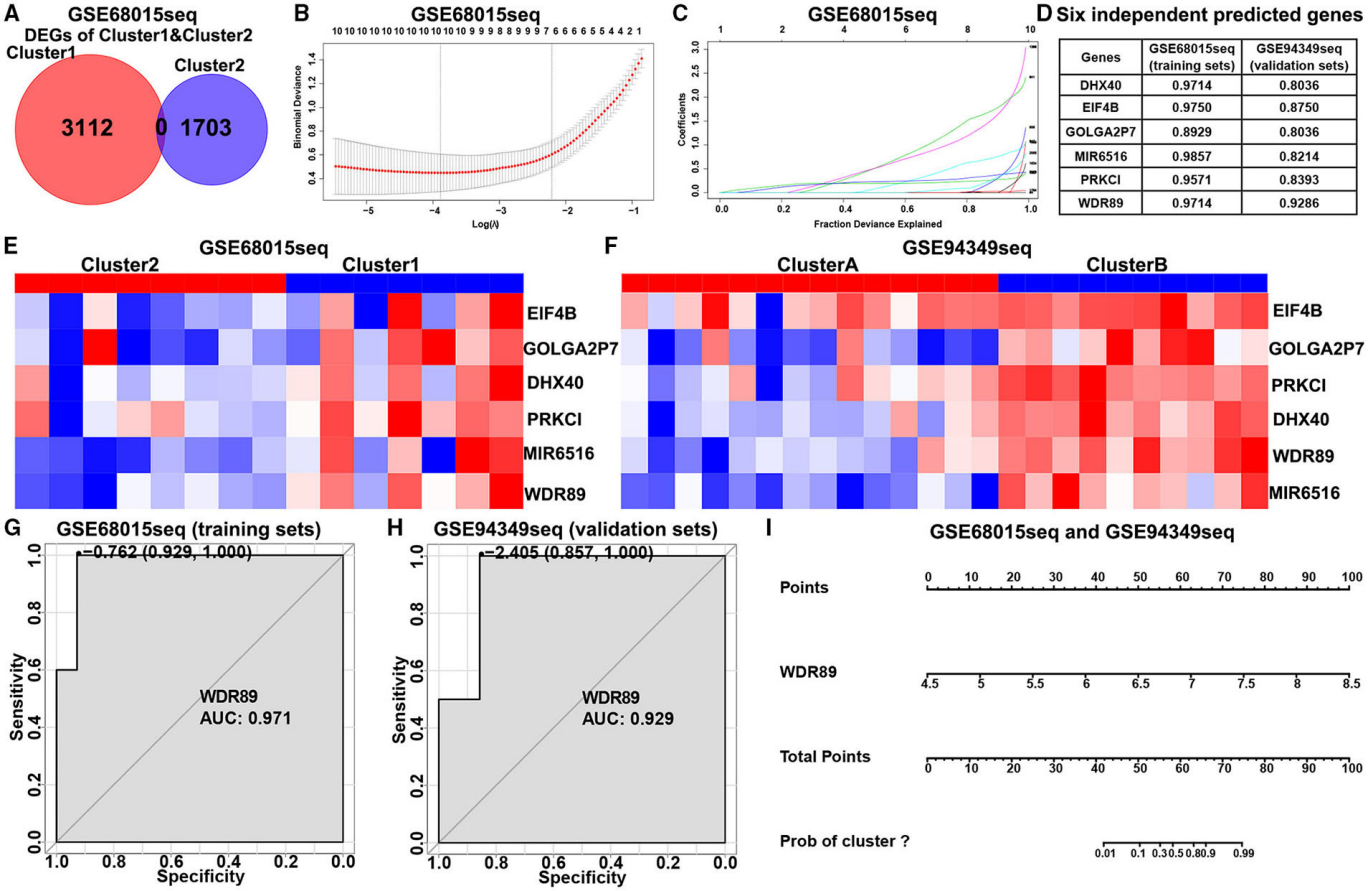
Construction of the classification prediction model. **(A)** Venn diagram showed the DEGs between-group cluster 1 and cluster 2 ACPs in the GSE68015 database. **(B)** LASSO coefficients profiles of 4,815 DEGs in the GSE68015 database. **(C)** LASSO regression with tenfold cross-validation obtained optimal prognostic genes using minimum lambda value. **(D)** Logistic analysis was used to evaluate the best six independent predicted genes. **(E,F)** Heatmap of the expression profiles of the six independent predicted genes between cluster 1/2 and cluster A/B in the GSE68015 and GSE94349 databases. **(G,H)** ROC curves showed WDR89-based predictive model could provide a more reliable classification assessment in the training sets (AUC = 0.971) and validation sets (AUC = 0.929). **(I)** WDR89-based nomogram model to predict the immune classification of ACPs in the GSE68015 and GSE94349 databases.

In the GSE60815 database, we identified 4,825 DEGs (3,112 upregulated genes of cluster 1 and 1,713 upregulated genes of cluster 2) for LASSO and logistic analysis and identified the best six independent predicted genes (WDR89, PRKCI, DHX40, EIF4B, GOLGA2P7, and MIR65161) (Figures 8A–F). A gene-based classification prediction model was constructed afterward. ROC curves showed that the WDR89-based predictive model provided a reliable classification assessment in the training sets (area under curves (AUC) = 0.971) and validation sets (AUC = 0.929) (Figures 8G,H). Finally, we developed a WDR89-based nomogram model to predict the classification of ACPs (Figure 8I).

### WDR89-Related Expression Profile in ACPs Relative to a Broad Range of Other Primary Pediatric and Adult Brain Tumor Types

**Figure 9:**
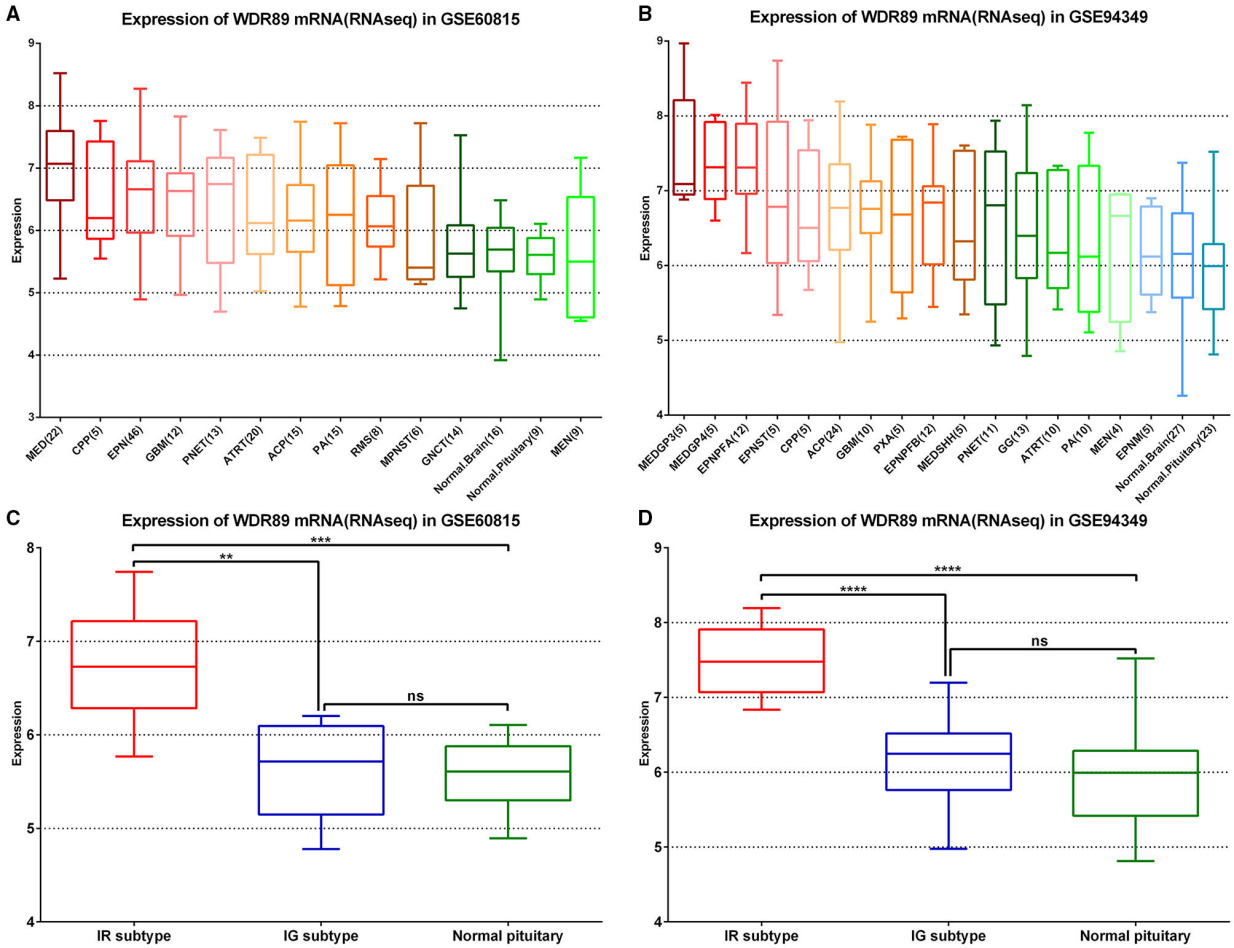
WDR89 related expression profile in ACPs relative to a broad range of pediatric and adult brain tumor types. **(A,B)** WDR89 mRNA expression in ACPs relative to a broad range of pediatric and adult brain tumor types in the GSE68015 and GSE94349 databases. **(C,D)** Expression of WDR89 in the IG and IR subtype of ACPs, and normal pituitary group. **p < 0.01, ***p < 0.001, ****p < 0.0001.

In the GSE60815 and GSE94349 databases, expression profile analysis suggested that compared with the normal brain group (including pituitary) and most other primary pediatric and adult brain tumors (including MEN, GNCT, MPNST, RMS, PA), WDR89 was highly expressed in ACPs (Figures 9A,B). In addition, the expression of WDR89 in the IR subtype of ACPs is higher than that in patients with the IG subtype of ACP (Figures 9C,D).

### GO and KEGG Analysis of WDR89-Associated Genes in ACPs

**Figure 10:**
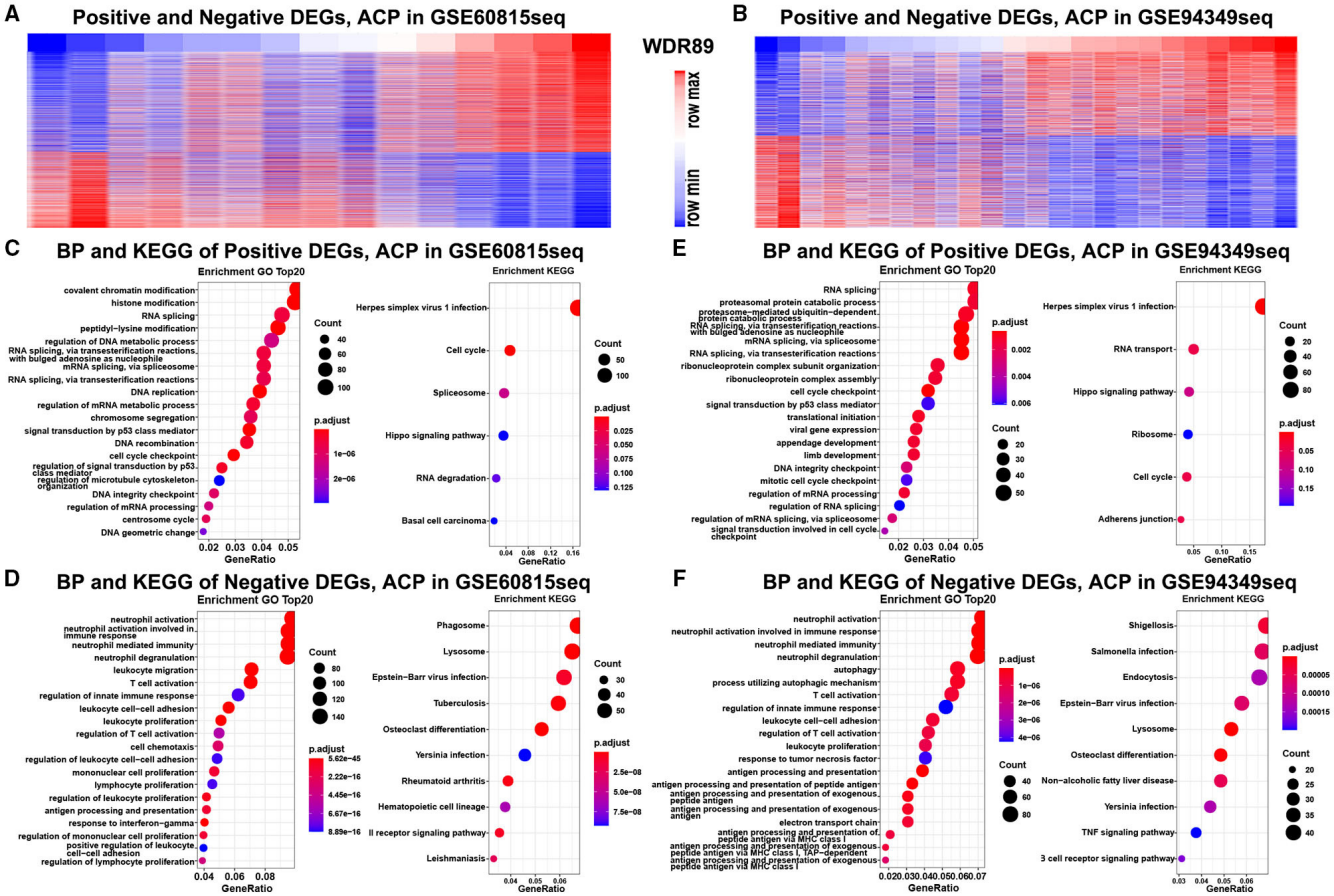
GO and KEGG analysis of WDR89-associated genes in ACPs. **(A,B)** Heatmap for genes most relevant to WDR89 generated by Pearson correlation analysis in the GSE68015 and GSE94349 databases. **(C–F)** GO and KEGG enrichment analysis of genes most relevant to WDR89.

Functional enrichment analysis demonstrated that genes negatively relevant to WDR89 (Pearson |*R*| > 0.5) were mostly involved in neutrophil activation, T cell activation, leukocyte proliferation, and TNF signaling pathway (Figures 10A,C,D). However, genes positively relevant to WDR89 were associated with RNA splicing, DNA replication, cell cycle, and Hippo signaling pathway (Figures 10B,E,F).

## Discussion

Cancer immunotherapy has completely revolutionized the treatment landscape of malignant tumors, which is a new type of treatment that has emerged after surgery, chemotherapy, radiotherapy, and targeted therapy (26, 27). Although cancer immunotherapy has been widely used in many tumors, there are still many challenges, such as limited efficacy and serious side effects (27, 28). Relevant studies have shown that only about 13% of patients could benefit from ICB therapy, and it is not yet possible to accurately determine which patients could benefit from immunotherapy (29).

Adamantinomatous craniopharyngiomas mostly have large cystic components. The rapid growth of the cystic component will compress and destroy the neighboring key structures. Therefore, the study of the pathogenesis of ACP cysts is particularly important. Up to now, studies have found that the expression of many inflammatory molecules in the cystic component of ACP are upregulated, such as alpha-defensins 1-3, IL6R, IL2RB, IL-1B, IL-6, CXCL1, CXCL8 (IL-8), IL-10, CXCR2, CXCL1 (GRO), IDO-1, IL-18, TNF, and IFNG (30–33). At the same time, related studies have also found that the expression of inflammatory molecules in the solid components of ACP is also upregulated, which further supports the important role of immune response in the pathogenesis of ACP (32, 34). A recent study found that immune checkpoint molecules PD-1 and PD-L1 are overexpressed in epithelial cell clusters in ACP (35), and these clusters of epithelial cells were found to play an important role in the growth of ACP (36–38). This research provides theoretical support for the treatment of ICB in ACPs.

Immune cells can induce excessive activation of intracellular signaling pathways or activation of abnormal signaling pathways by secreting proinflammatory factors and chemokines, and ultimately promote tumor proliferation, invasion, and metastasis (39, 40). A related study found that there was a large number of immune cell infiltrations between ACPs and important structures such as the hypothalamus, and there is also tight adhesion formation. The degree of inflammatory response is significantly positively correlated with the incidence and severity of the hypothalamus–pituitary deficiency (41). Therefore, we inferred that the inflammatory response between the tumor and important structures may cause the difficulty of tumor dissection during the operation, which may lead to the occurrence of serious postoperative complications and tumor recurrence. The inflammatory response may be one of the important factors for the worse prognosis of ACPs.

In summary, it has been discovered and verified that many inflammatory molecules and cytokines in ACP are overexpressed, and the IME of ACP plays an important role in the development of tumors (32). Therefore, cancer immunotherapy, such as ICB therapy, is a promising therapy for ACPs, but there is still a long way to go to fully explain the potential of immunotherapy in ACPs.

In this research, we summarized the immune profile of ACP and identified two novel immune subtypes (namely IR subtype and IG subtype), which showed completely different immunotherapy responsiveness rates. Compared with the IR subtype, the IG subtype is involved in various inflammatory and immune responses. Simultaneously, the expression of immune checkpoint molecules in the IG subtype is higher than that of the IR subtype, and the IG subtype showed a better response to immunotherapy. We also constructed a WDR89-based model to predict the immune classification of ACPs with excellent performance. The related study demonstrated that the degree of inflammatory response is significantly positively correlated with the incidence and severity of the hypothalamus–pituitary deficiency (41). Therefore, this subtype of ACPs is in urgent need of immunotherapy. However, the lack of *in vitro, in vivo*, or clinical validation of these findings is a major limitation in this study. In the future, our team will work on an in-depth analysis of the IME of ACPs through *in vitro* and *in vivo* methods, and provide theoretical and practical support for the application of immunotherapy in ACPs.

## Data Availability Statement

Publicly available datasets were analyzed in this study. This data can be found at: National Center for Biotechnology Information (NCBI) Gene Expression Omnibus (GEO), https://www.ncbi.nlm.nih.gov/geo/, GSE60815 and GSE94349.

## Author Contributions

FY: conceived the project. CM and FY: supervised the project. FY, XC, and JZ: downloaded the data and performed the statistical analysis. CM, FY, LY, YW, CT, and ZC: interpreted the results. All listed authors read and approved the final manuscript.

## Conflict of Interest

The authors declare that the research was conducted in the absence of any commercial or financial relationships that could be construed as a potential conflict of interest.

## Publisher's Note

All claims expressed in this article are solely those of the authors and do not necessarily represent those of their affiliated organizations, or those of the publisher, the editors and the reviewers. Any product that may be evaluated in this article, or claim that may be made by its manufacturer, is not guaranteed or endorsed by the publisher.
